# sensaas: Shape‐based Alignment by Registration of Colored Point‐based Surfaces

**DOI:** 10.1002/minf.202000081

**Published:** 2020-06-23

**Authors:** Dominique Douguet, Frédéric Payan

**Affiliations:** ^1^ Université Côte d'Azur Inserm, CNRS, IPMC 660 route des lucioles 06560 Valbonne France; ^2^ Université Côte d'Azur CNRS, I3S, Les Algorithmes - Euclide B 2000 route des lucioles 06900 Sophia Antipolis France

**Keywords:** Shape-based alignment, molecular surfaces, point clouds, registration, molecular similarity

## Abstract

sensaas is a tool developed for aligning and comparing molecular shapes and sub‐shapes. Alignment is obtained by registration of 3D point‐based representations of the van der Waals surface. The method uses local properties of the shape to identify the correspondence relationships between two point clouds containing up to several thousand colored (labeled) points. Our rigid‐body superimposition method follows a two‐stage approach. An initial alignment is obtained by matching pose‐invariant local 3D descriptors, called FPFH, of the input point clouds. This stage provides a global superimposition of the molecular surfaces, without any knowledge of their initial pose in 3D space. This alignment is then refined by optimizing the matching of colored points. In our study, each point is colored according to its closest atom, which itself belongs to a user defined physico‐chemical class. Finally, sensaas provides an alignment and evaluates the molecular similarity by using Tversky coefficients. To assess the efficiency of this approach, we tested its ability to reproduce the superimposition of X‐ray structures of the benchmarking AstraZeneca (AZ) data set and, compared its results with those generated by the two shape‐alignment approaches shaep and shafts. We also illustrated submatching properties of our method with respect to few substructures and bioisosteric fragments. The code is available upon request from the authors (demo version at https://chemoinfo.ipmc.cnrs.fr/SENSAAS).

## Graphical abstract

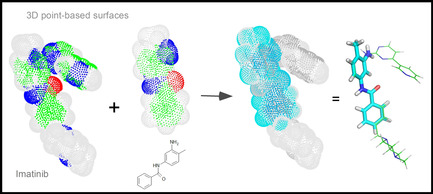

## 1 Introduction

3D shape is an important molecular feature in biological activity.^1^ Over years, a wide range of 3D shape similarity methods have been developed to compare and quantify the similarity between molecules. Molecular shape comparison methods can be classified according to the representation used. *Atomic‐distance* based methods are alignment‐free algorithms that describe the shape by using inter‐atomic distance distributions. An example is the Ultrafast Shape Recognition (USR) tool developed by Ballester and Richards, which encodes the information into a string vector.^2^ *Atom‐centered Gaussians* based methods, including the program ROCS, are probably the most widely used to evaluate the maximum volume overlap between two molecules.^3^, ^4^ A third popular class uses *molecular fields* to describe, for example, the electrostatic or the hydrophobic potential, calculated at points of a grid around the molecule. In their review of 2018, Kumar and Zhang summarized several variations and hybridizations of these main classes.^5^ For example, shaep is a tool that uses both *molecular fields* and *Gaussian functions* representations to estimate the molecular similarity^6^ while shafts is an algorithm that combines a pharmacophore matching method and *Gaussian functions*.^7^ Alternative classes of shape similarity methods are *surface‐based* approaches that use various representations of the surface such as spherical harmonics (parafit,^8^ shems 9 and hpcc 10) and their extensions (3D Zernike descriptors^11^), probability distribution histograms as shape signature,^12^ alpha shapes as coarse representations of the surface^13^, ^14^ and point‐based surfaces. Point‐based surface methods describe the molecular surfaces with sets of 3D points, clustered to form similar surface regions also called patches. Local surface properties of the surface patches are then extracted to represent the shape. Goldman and Wipke use quadratic shape descriptors of a subset of points called critical points to evaluate pairings between small sections of the surfaces.^15^ Cosgrove et al.,^16^ Exner et al.,^17^ Hofbauer et al.,^18^ Baum et al.^19^, ^20^, ^21^ and Krotsky et al.^22^ also developed approaches that divide the molecular surface into a set of patches enclosing an area of similar properties. Then, clique detection algorithms are usually used to identify maximal common subgraphs and to perform a rigid‐body superimposition between two sets of patches. Clique detection is a combinatorial problem known to be NP‐hard and, thus, cliques are impracticable when one wants to work with a large number of points. In the current work, we describe a point‐based surface method which aligns thousands of colored points by using registration methods, initially developed for matching dense 3D point clouds acquired by 3D sensors.

Representing a molecular surface by using a 3D point cloud is not a new concept.^1^, ^5^, ^23^, ^24^, ^25^ A point cloud has this advantage of being an intuitive description, suited for visualization and for building a mental model. For example, this representation highlights which atoms contribute to the surface and, thus, may interact with its surroundings. By contrast, working with 3D graphs do not allow to distinguish atoms that are masked by others. Another feature of point‐based surfaces is the capacity to associate some extra attributes to the points such as color labels according to the nature of the underlying atoms. In their review, Kumar and Zhang pointed out that surface‐based shape similarity methods are a particular class of shape similarity methods that are still in infancy.^5^, ^24^ Indeed, although they underlined some particular interesting features of surface‐based methods such as comparing shape of ligands with that of binding pockets, they indicated that their current concerns are the slowness, an efficiency lower than Gaussian overlay‐based shape similarity methods and the inability to identify local similarities. In our study, we show that a surface‐based shape similarity method can now run reasonably fast and is able to identify global and local similarities.

3D point set registration, point set matching or geometric registration refer to the same class of methods that aim to align several sets (or clouds) by identifying nearest‐neighbor correspondences and minimizing the point‐pair distances.^26^, ^27^ The most popular approach is the ICP (Iterative Closest Point) method which gave rise to numerous variants. Point set registration is a fundamental problem in many domains: pattern recognition, computer vision or data reconstruction. Today, 3D registration methods that use geometrical features such as curvature, surface normals or higher order local descriptors are extensively used.^28^, ^29^, ^30^

Registration is often considered as a hard optimization problem because perfect point‐to‐point correspondences rarely exist: the point clouds might partially overlap only, or the underlying objects may have particular local geometrical features. Nevertheless, we show in this paper that 3D point set registration can be particularly attractive to align molecular shapes and sub‐shapes. The resulting approach is called sensaas, for sensitive surface as a shape. The following sections detail the sensaas workflow and show its ability to reproduce experimentally verified superimpositions of dissimilar molecules of the benchmarking AstraZeneca (AZ) data set.^31^ Originally, this data set was created for validating pharmacophore programs but it also provides an appropriate benchmarking data set for evaluating the general applicability of a molecular alignment method.^32^ To assess the accuracy of alignments provided by sensaas, we compared them with those generated by the state of the art methods, shaep and shafts. Finally, the submatching property of sensaas is emphasized by aligning some molecules of different sizes such as substructures and fragments.

## 2 Material and Methods

### 2.1 Sensaas Workflow

**Figure 1:**
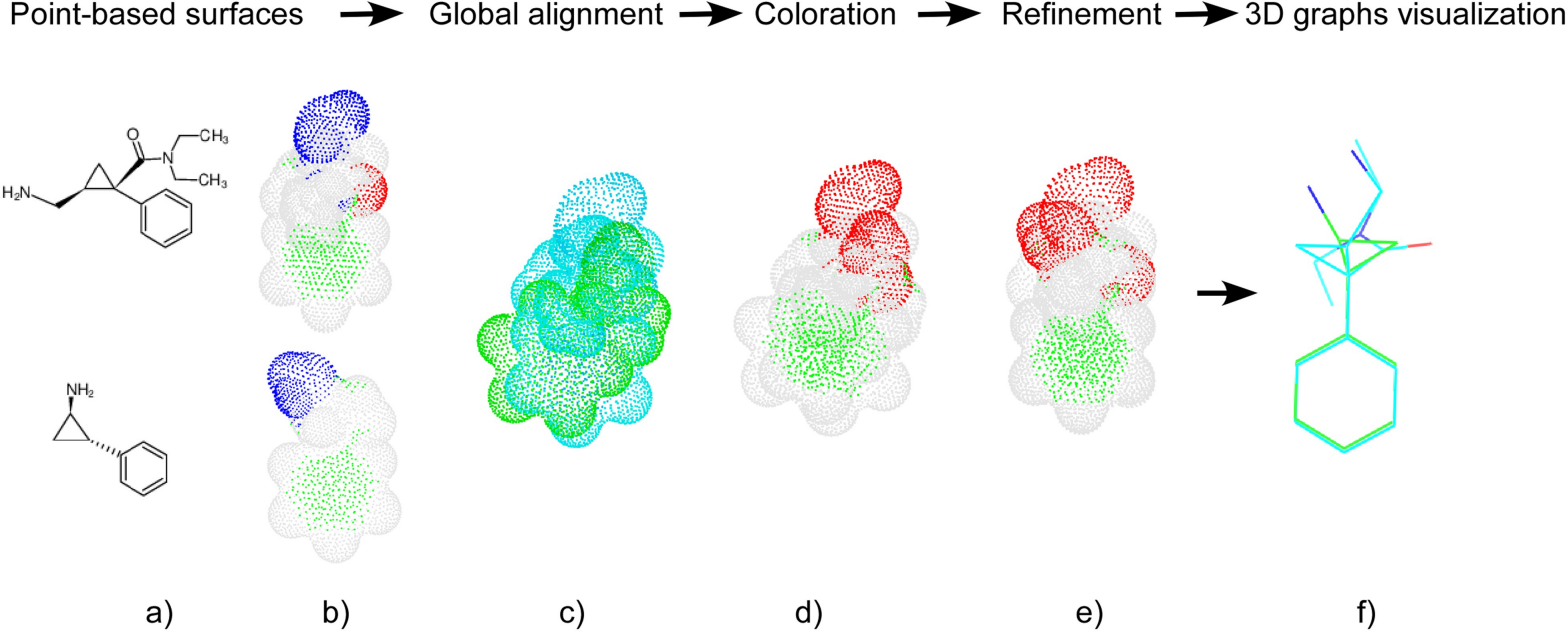
Schematic representation of sensaas workflow for matching two molecules. a) Input molecules; b) Point‐based representation of the molecular surfaces; points are colored according to the nature of the closest atom; c) Global superimposition (geometry‐aware only); Point clouds are colored in blue and green for visualization purpose; d) Same global superimposition but colored according to the physico‐chemical classes; e) Local refinement (geometry‐ and color‐aware); f) Final alignment of the corresponding 3D graphs.

An overview of sensaas is illustrated in Figure 1. It can be divided into four stages as follows. Let us consider two molecules that we want to align:

Step 1. Generate a 3D point cloud of the molecular surface for each molecule. Each point is described by its coordinates in 3D space (geometry) and a color according to the nature of the underlying atom (Figure 1b).

Step 2. Initialize the alignment. Point clouds are globally superimposed by finding the best matching in terms of geometry only (Figure 1c).

Step 3. Refine the initial alignment. The initial superimposition is locally refined by taking into account the geometry but also the physico‐chemical properties mapped on the surface (pharmacophore features for example). This is done by i) defining classes of properties; ii) coloring each point according to its class (Figure 1d); iii) refining the initial superimposition by finding the best matching between these two colored point clouds (Figure 1e). This step results into a transformation matrix to translate and rotate the moving molecule.

Step 4. Calculate scores and display the superimposition. Fitness scores are calculated using point clouds and the final alignment of the 3D graphs is achieved by applying the transformation matrix to the 3D graph of the moving molecule (Figure 1f).

Each stage is described in detail in the next subsections.

*a) Generation of 3D point clouds*. Let us consider two molecules *M*_1_ and *M*_2_ to align. The goal of this step is to generate point‐based 3D representations *S*_1_ and *S*_2_ of the molecular surface of *M*_1_ and *M*_2_, respectively. *S*_1_ and *S*_2_ result from the computation of the van der Waals (vdW) surface of each molecule by using the program developed by Eisenhaber et al.,^33^ with van der Waals radii taken from A. Bondi.^34^ Finally, *S*_1_ and *S*_2_ consist in two sets of 3D points uniformly distributed on the vdW surface. Each point is depicted by its position in 3D space (x, y and z) and a label indicating the type of its closest atom.

*b) Geometry‐based alignment*. Considering two point sets, *Source* and *Target*, a rigid registration (or matching) consists in finding a transformation matrix T describing how to rotate and translate the *Source* in order to be superimposed to the *Target*. In our context, we have no *a priori* information about the initial pose of the point sets in 3D space, and about the existence of overlapping areas between the molecular surfaces. Therefore, we use a two‐stage procedure: i) perform a global registration to obtain an initial “coarse” superimposition, and ii) refine the initial alignment by a local registration. This local registration, that would be inefficient without applying the global one, is explained in the next section.

**Figure 2:**
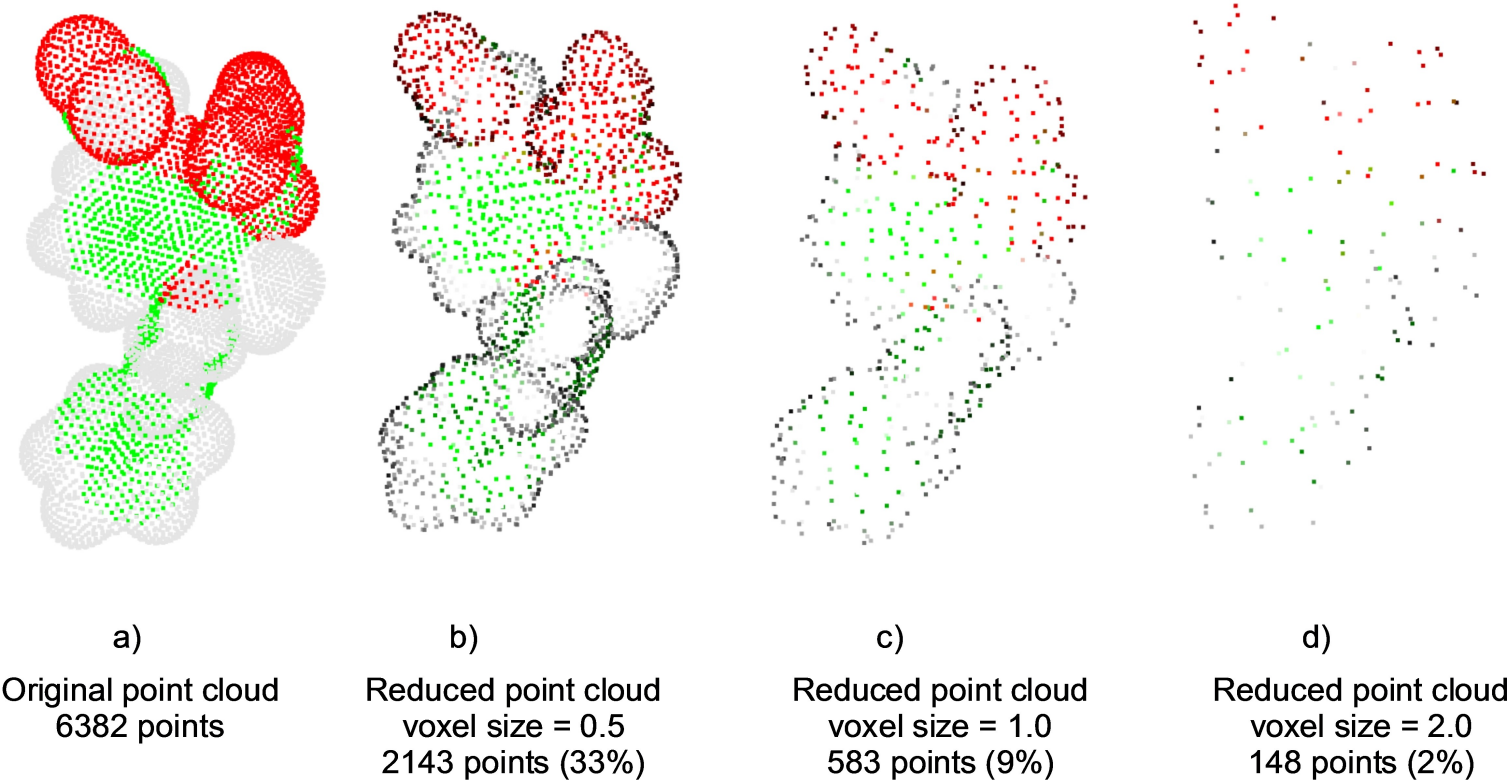
Effect of the voxel size (VS) on the number of points in the reduced point cloud for the molecule me‐indoxam.

To match 3D point sets globally, it is generally accepted that it is more efficient to match local 3D descriptors computed on a limited number of points rather than to match the points themselves. Local 3D descriptors provide pose invariant local geometrical properties of the surface inherent to a point cloud. In sensaas, we use Fast Point Features Histograms (FPFH) proposed by Rusu et al.^35^ Such a histogram is defined by a 33‐dimensional vector. In a nutshell, according to the shape of a histogram, one can know if the surface around a point looks like a plane, a cavity, a hump… As reduced sets of points on which the FPFH are calculated, we use simplified versions of *S*_1_ and *S*_2_, called S*_1_ and S*_2_. S*_1_ and S*_2_ are simply obtained by subdividing the bounding box of *S*_1_ and *S*_2_ into a set of small cubes, named voxels hereinafter. All points in a voxel are removed except the closest point to the centroid. This process is also referred to as down‐sampling the point cloud. Figure 2 displays the reduced point clouds obtained for different voxel sizes (VS) for the example molecule me‐indoxam.

A FPFH is computed for each point of the reduced point clouds. It results in two finite sets of FPFH. Then, the RANSAC algorithm is used to match them.^36^ RANSAC is an iterative model fitting framework widely used. At each iteration, it consists in picking N points from the reduced cloud S*_1_, and in identifying N *corresponding points* in the cloud S*_2_, by querying their nearest neighbor in the FPFH space. At this step, it is possible to compute the transformation matrix T, to align S*_1_ and S*_2_. Before that, two checks are carried out to make sure the suggested transformation is meaningful in the 3D space.

- The first test estimates the proximity of points by applying T on the N points of S*_1_, and checking that the distance between these points and their corresponding points in S*_2_ is lower than a given threshold (VS*1.5).
- The second test checks that inter‐distances (edge lengths) between the N points of S*_1_ are close to the inter‐distances between the N corresponding points in S*_2_. This is considered as satisfactory if each of them does not differ by more than 10 %.

If these two tests are successful, the matching at this iteration is validated, the transformation matrix is applied to S*_1_ and, a RMSE value (Root Mean Square Error) is computed between it and S*_2_. The maximal number of querying is 400000 but if tests are successful 1000 times, the procedure stops, the alignment with the smallest RMSE value is selected, and the associated transformation matrix T is applied to the entire point cloud *S*_1_. As a result, we obtain the point cloud *S*_1_^g^ globally aligned on the other one (Figure 1c). From now on, this initial superimposition step is called global.

*c) Local refinement based on geometry and physico‐chemical properties*. In many cases, an alignment obtained with a “global” approach is perfectible and can be improved by a “local” alignment.

3D shape is an important feature in molecular recognition but the distribution of physico‐chemical properties in space as it is represented by pharmacophore features is determinant for the binding affinity of ligands. Such characteristics can be conveniently used to improve the alignment of molecules, by optimizing the matching of properties along with the geometry. To reach this objective, we chose to use an ICP variant for colored point clouds developed by Park et al.^37^ The interest of this approach is to obtain a tighter alignment by matching both geometry and color of points. This method is particularly appropriate for our molecular surfaces as each point has a label that can be seen as a coloration. We indicated in *(a)* that each input point is described by its position in 3D space (x, y and z) and its label indicating the type of its closest atom. We categorize atom types into four classes that will be seen as colors during this stage. Examples of colored clouds resulting from this classification are visible in Figure 1d. In our implementation, labels aim to recapitulate typical pharmacophore features such as aromatic, lipophilic and polar groups.^38^, ^39^

The first class includes non polar hydrogen (H) and halogen atoms excepting fluorines (Cl, Br and I). Hydrogen and halogen atoms are molecule endings. They are the most frequent atoms that contribute to the surface geometry and coloration, and thus, highlight the apolar surface area. Points belonging to this class are colored in white in our study (Figure 1d).
The second class includes polar atoms able to be involved in hydrogen bonds such as N, O, S, H (if linked to N or O) and F.^40^ Points belonging to this class are colored in red in our study (Figure 1d).
The third class includes “skeleton elements” such as C, P and B. Points belonging to this class are colored in green in our study (Figure 1d). This class sometimes contributes to the surface. For example, aromatic carbons may contribute to the surface by exposing a typical pattern formed by two parallel green patches as shown in Figure 1d. On the contrary, a sp3 carbon does not contribute significantly to the surface because hydrogen atoms that are linked to it masked it.
The fourth class includes all elements not listed in the first three classes. This class is empty for most small organic molecules in medicinal chemistry. Points belonging to this class are colored in blue in our study.

Globally, ICP consists in i) selecting a set of points in the clouds *Source* and *Target*; ii) matching the selected points in *Source* with the “optimal” corresponding points in *Target* (historically the “closest points”); iii) estimating the transformation matrix T that gives the best alignment of *Source* and *Target*, by minimizing iteratively a cost function E depending on the corresponding points; iv) transforming *Source* with T, and going back to ii) until reaching convergence, or a maximum number of iterations.

The ICP variant for colored point clouds^37^ employed in sensaas uses a cost function E that takes both geometric and color information for alignment: *E* (*T*)=*δ E*g (*T*)+(1−*δ*) *Ec*, where *δ*, set to 0.8, balances the influence of the two terms. Eg is the objective function proposed in the *point‐to‐plane* ICP variant.^29^ The last matches the points by a procedure called “normal shooting” from *Source* to *Target* (i. e. projection in the normal direction), and then determines the matrix T that minimizes the RMSE between the corresponding pairs. *Ec* is the term that weights the alignment of points of same color. It measures the difference between the color of the *Source* points and the color of their projection on the tangent plane to their corresponding *Target* points. To summarize, the local registration stage of sensaas consists in:

- taking as input the two point clouds aligned by global (*S*_1_^*g*^ and S_2_);
- matching the reduced point clouds S*_1_^*g*^ and S*_2_;
- applying this ICP variant and stopping when the number of iterations reaches 100, or when the relative RMSE between two iterations is lower than 1e–6.

As output, we obtain the final transformation matrix to superimpose *S*1 to *S*2. From now on, this local registration stage is called color.

### 2.2 Alignment Evaluation

To evaluate alignments, it is common to compute RMSE values and fitness scores. A RMSE indicates how close the points of two aligned clouds are paired whereas a fitness indicates how many points are paired. Points are considered paired if their distance is lower than a given threshold. In our implementation, we set the threshold value to 0.3 because it is the average distance between two adjacent points in our original point clouds. The fitness equals 1 if the alignment is perfectly achieved for two identical 3D molecular structures. Increasing this value would artificially increase the fitness by pairing more distant points. Fitness is also essential to calculate how many points with similar colors finally match. We defined three different fitness scores:

– *gfit* is the ratio between the number of points of the transformed *Source* that match points of the *Target*, and its total number of points:

(1) $$gfit=\frac{number-of-matching-points-in-Source\;}{Total-number-of-points-in-Source\;}\;$$

– *cfit* is the sum of the fitness for each class, to specifically evaluate the matching of the colored points (k=4 classes):

(2) $$cfit=\;\frac{\sum_{i=1}^{k}nb-matching-points-in-Source-for\;class-i}{\sum_{i=1}^{k}number-of-points-in-class-i}$$

– *hfit* is the sum of the fitness for each class except the first class, to specifically evaluate the matching of polar and aromatic points (classes 2, 3 and (3):

(3) $$hfit=\;\frac{\sum_{i=2}^{k}nb-matching-points-in-Source-for\;class-i}{\sum_{i=2}^{k}number-of-points-in-class-i}$$

Those scores are similar to a Tversky coefficient^41^ tuned to evaluate the embedding of one object into another one, here the molecules *Source* and *Target*. This implies that scores calculated with respect to *Target* may differ from those calculated with *Source*. Moreover, the smallest point cloud of the two will always obtain the highest fitness score as more points are paired, proportionally. In the following, “global *gfit*” relates to the (intermediary) fitness value calculated after global and terms *gfit*, *cfit* or *hfit* means that the whole registration algorithm (global+color) is applied before calculating fitness values.

### 2.3 Choice of the Voxel Size (VS)

To be efficient in a context of molecular surfaces, we had to tune the voxel size (VS) for global and color. All the functions used in global and color are implemented with functions of the open‐source library Open3D0.7.0.^42^ As described in the workflow description, the VS influences the number of points in the reduced point cloud. It is important to select a value consistent with the density of the point cloud. In our study, points are uniformly distributed on the vdW surface and the distance between two adjacent points is ∼0.3 Å (NSC program (version 2.0) from Eisenhaber et al.^33^). Table 1 shows the average percentage of remaining points for various VSs. As expected, a VS≤0.3 does not significantly lower the number of points since more than 70 % of initial points are kept. On the contrary, a VS≥1 leads to the removal of more than 90 % of initial points. If too many points are removed, the matching fails. To select an appropriate VS value, we analyzed pairwise alignments for VS values ranging from 0.2 to 1.2 (*i. e*. from 95 % to 5 % of remaining points) with an increment of 0.1.

**Table 1:** Percentage of remaining points after reducing a point cloud.

Voxel size	Percentage of remaining points
0.1	99–100
0.2	95–96
0.3	67–70
0.4	43–46
0.5	29–31
0.6	21–22
0.7	16–17
0.8	12–13
0.9	10
1.0	8
1.1	7–8
1.2	5–6
3	0.9–1.1
7	0.3–0.6

**Figure 3:**
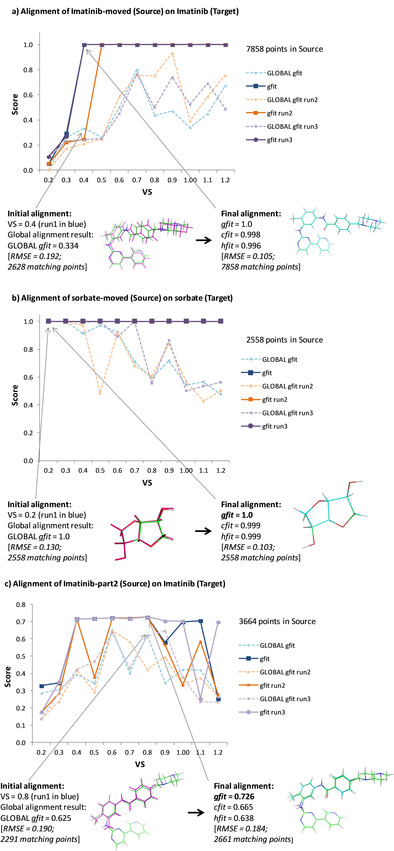
*gfit* versus global *gfit* in function of VS. Three runs are plotted (blue, orange and mauve lines). The initial and final alignments are displayed with their fitness scores, number of matching points and RMSE values. The *Source* 3D graph aligned with global only is colored in magenta (at left). *Target* and aligned *Source* 3D structures are colored in green and cyan, respectively (at right). (a) Imatinib/Imatinib‐moved pair. (b) Sorbate/sorbate‐moved pair. (c) Imatinib/Imatinib‐part2 pair.

In the following examples, we studied self‐matchings of molecules sorbate and Imatinib with rotated and translated versions of themselves (named “sorbate‐moved” and “Imatinib‐moved”, respectively). In such experiments, the perfect superimposition is expected, and *gfit* values must equal 1. We also assessed the substructure matching of Imatinib with a part of itself, Imatinib‐part2 (Figure S1a and S1b in the Supporting Information). Figure 3 displays resulting alignments, and *gfit* scores plotted in function of VS. First, initial alignments obtained with global are always improved by color (for all runs and any VS): *gfit* is always higher than global *gfit* (Figure 3a, b and c), and the final RMSE is always lower than the intermediary RMSE obtained by global. Second, results show that there is no single value of VS for which the best solution could be found for all cases. For example, when VS equals 0.2 or 0.3, perfect superimpositions are observed for the sorbate case, but not for Imatinib. In the substructure matching (Figure 3c), global *gfit* and *gfit* scores vary from one test to another, but good alignments and scores are obtained when VS is 0.4, 0.6, 0.7 or 0.8. In that case, VS values larger than 0.8 lead to more erratic results. In conclusion, VS strongly influences the accuracy of the alignments, and a unique and optimal value cannot be known *a priori*. Thus, as the down‐sampling is reproducible for a given point cloud and for a given VS, we finally chose to successively execute eleven alignments with VS ranging from 0.2 to 1.2 with an increment of 0.1 and, to keep the best alignment. We consider that an alignment is good if it has a good shape overlap (high *gfit*) and a good superimposition of common pharmacophoric features (high *hfit*). Thus, the alignment with the largest sum of *gfit* and *hfit* values is considered as the best one.

### 2.4 Computing Time

**Figure 4:**
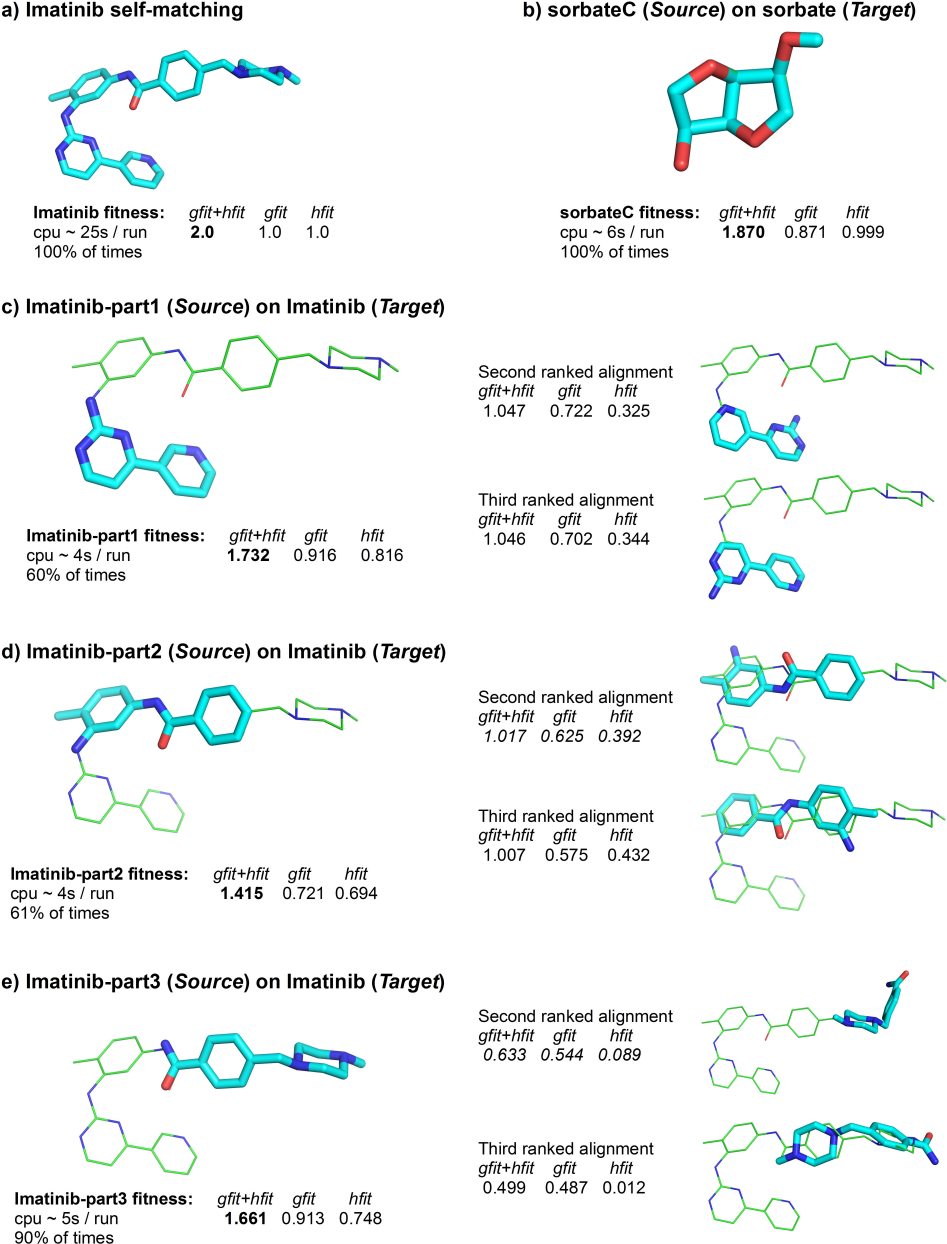
Results of self‐ and substructure‐matchings. Each run was performed 100 times with a random orientation for the *Source* each time. a) Imatinib self‐matching. b) sorbateC (*Source*) on sorbate (*Target*). c) Imatinib‐part1 (*Source*) on Imatinib (*Target*). d) Imatinib‐part2 (*Source*) on Imatinib (*Target*). e) Imatinib‐part3 (*Source*) on Imatinib (*Target*). Corresponding superimposition(s) of 3D graphs, the percentage of times, the mean CPU time and the scores *gfit*+*hfit*, *gfit* and *hfit* are indicated. *Target* 3D structures are colored in green and *Source* 3D structures are colored in cyan. Hydrogen atoms are hidden in 3D graphs.

All experiments have been carried out on a Dell PowerEdge R940 (Intel Xeon Gold 5220). sensaas is running using the Debian version 5.3.15‐1 of Linux. The processing time for molecules used in present tests takes between 3 to 25 seconds per run (Figure 4). Computation times of 25 seconds occur when a molecule is aligned on a shifted version of itself, for example in self‐matching tests. However, aligning a molecule on itself is a rare and specific experiment, per se. In our study, we only used it as a control. No optimization of the code was carried out so far but an optimization of the point cloud preprocessing and the use of Python threads over voxel size could bring a substantial improvement in computation times.

### 2.5 Data Visualization

In this study, the Open3D function *visualization.draw_geometries* and the software PyMOL (version 1.3) were used to visualize alignments and generate illustrations.

### 2.6 sensaas Validation with Self‐ and Substructure Matchings

Because of the stochastic nature of the registration methods used in sensaas, we carried out an analysis of its reproducibility. In studied examples, experiments were repeated one hundred times, and resulting superimpositions and fitness scores were examined. Figure S1 in the Supporting Information displays the structures of molecules: the previously analyzed sorbate/sorbate‐moved and Imatinib/Imatinib‐moved pairs, and four substructures pairs sorbate/sorbateC, Imatinib/Imatinib‐part1, Imatinib/Imatinib‐part2 and Imatinib/Imatinib‐part3, whose resulting alignments are also easy to validate.

### 2.6 Benchmarking AZ Data Set

Tests involving self‐ and substructure matchings are prerequisites but are not broad enough to evaluate the general applicability of a molecular alignment method. The only experimental evidence of alignments between different molecules is their co‐crystallization with the same protein. Therefore, the performance of sensaas was assessed by comparing its alignments using the 121 experimental overlays of the AZ test set available at the CCDC Center.^31^ This data set contains 1465 co‐crystallized ligands extracted from the Protein Data Bank (PDB). The authors annotated overlays according to shape, feature and 2D fingerprint similarities between ligands and, finally classified overlays as either easy (22 cases), moderate (73 cases), hard (18 cases) or unfeasible (8 cases) to predict.^43^ For each of the 121 proteins, sensaas was run using each experimental ligand as the *Target* in turn. Then, each resulting pose of other ligands (*Source*) was compared to their experimental pose and scored with the heavy atom root‐mean‐square deviation (RMSD). It is usually accepted that a pose is correctly predicted if the RMSD is below 2 Å. Results were then compared to those obtained with shaep and shafts.

### 2.7 Fragment Alignments

As we aim at developing a method that performs well in scaffold hopping and bioisostere replacement (*i. e*. replacing one chemical group by another one with a different chemotype),^44^, ^45^ we evaluated the sensaas ability to align the bioisosteric fragments tetrazole and carboxylate.^46^, ^47^ Such submatching properties requires that sensaas must not only align molecules of the same size successfully, but also align substructures and bioisosteric fragments correctly, even if they are small. To this end, we selected the drug Valsartan which contains both a carboxylic acid and a tetrazole ring (Figure S1d–f). Results were compared to those obtained with shaep and shafts.

## 3 Results and Discussion

### 3.1 sensaas Validation with Self‐ and Substructure Matchings

Figure 4 shows results of pairwise alignments for self‐ and substructure‐matchings that are easy to validate. Because of the stochastic nature of the registration methods, those tests were repeated 100 times to identify possible alternate alignments. For the self‐matching of Imatinib, the perfect alignment is always found (Figure 4a). Similarly, the sorbate/sorbateC alignment is a trivial test that sensaas successfully achieves 100 % of times (Figure 4b). Regarding substructure matchings, the best, expected, alignment is found most of the times and ranked first (Figure 4c, d and e, alignments at left). Over the repeats, alternate alignments are generated but their fitness scores are always lower (Figure 4c, d and e, alignments at right). We then assessed the ability of sensaas to generate the expected alignment when a different conformation of the *Source* is used. Figure S2a in the Supporting Information shows the best alignment of such a conformer of the *Source* Imatinib‐part3 that has a RMSD of 1.99 Å with the original substructure conformation. This alignment shows the expected matching of the aromatic and piperazine rings, but an inversion of the amide function. This is caused by the conformer that differs from the original Imatinib structure, precluding a perfect matching. Therefore, moderate variations in conformation do not prevent the identification of the correct alignment by sensaas. We further investigated the capacity of sensaas to identify the best Imatinib‐part3 conformer from a library of 74 conformers generated with RDKit Open‐Source Cheminformatics Software (http://rdkit.org).^48^ Figure S2b and S2c in the Supporting Information displays score values for the 74 conformers and the first ranked superimposition, respectively. Conformer number 36 is the first ranked solution and is perfectly aligned with the Imatinib structure. This result shows that aligning a library of conformers with sensaas succeeds in identifying the conformer allowing the best alignment.

### 3.1 Benchmarking AZ Data Set

**Figure 5:**
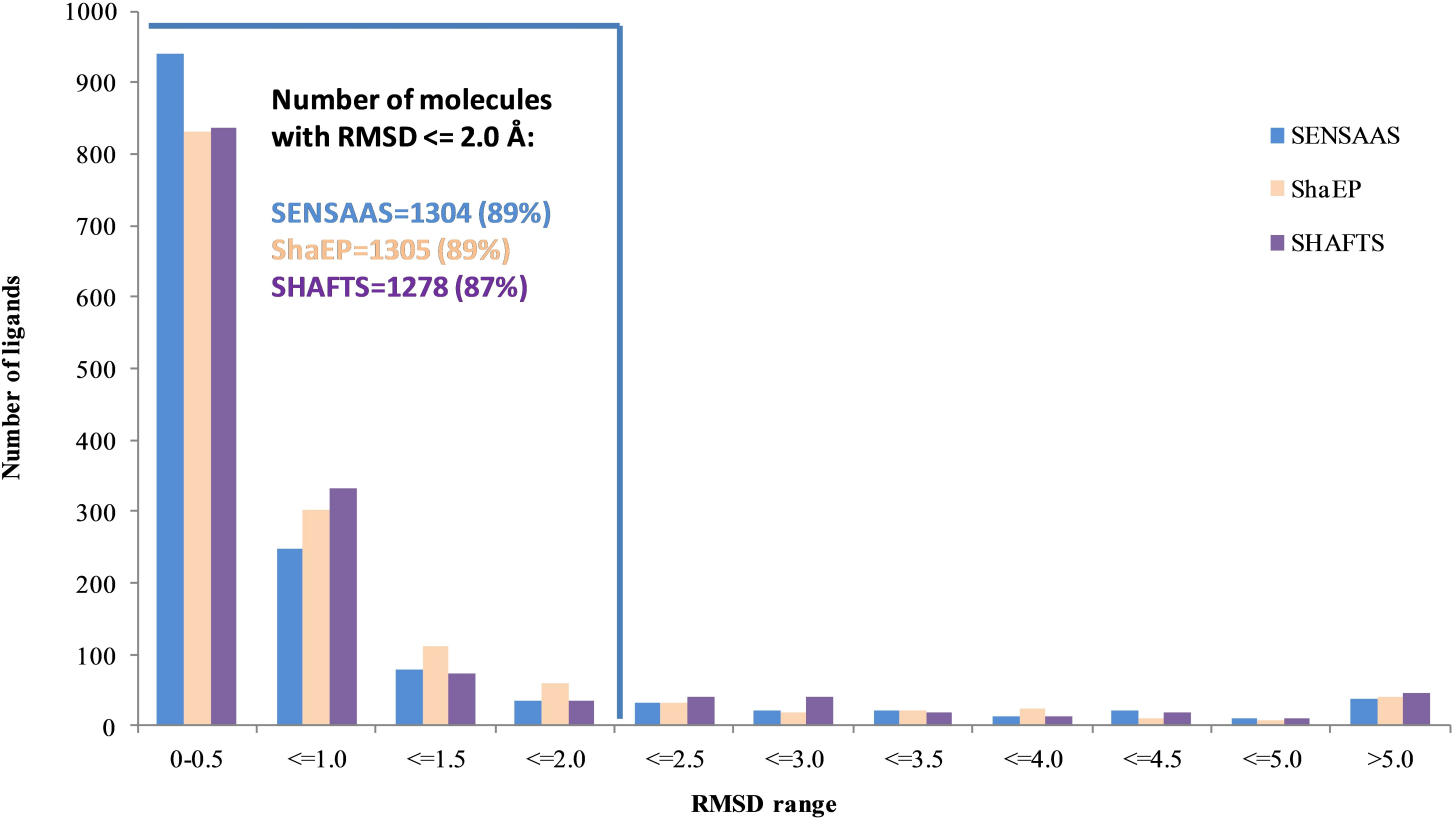
Distribution of the lowest RMSD obtained for each molecule of the AZ data set (1465 ligands). sensaas results are colored in blue, shaep results are colored in beige and shafts results are colored in mauve. Inset: a large majority of molecules are successfully reproduced with a RMSD≤2.0 Å.

As the only experimental evidence of alignments between different molecules is their co‐crystallization with the same protein, we tested the ability of sensaas to reproduce the superimposition of X‐ray structures of the benchmarking AZ data set. Then, to assess its performance, we compared results with those generated with shaep 6 and shafts.^7^ In this experiment, sensaas was run ten times and the best ranked solution, *i. e*. best *gfit*+*hfit* value, was retained. shaep and shafts, being deterministic methods, were run once with the default settings and with partial charges calculated with cxcalc calculator.^49^ Contrary to shaep and shafts, sensaas does not require the calculation of partial charges. Figure 5 shows the distribution of the lowest RMSD obtained for the 1465 molecules. The three methods produce comparable distributions with small differences within each interval in favor of one method or the other. The mean RMSD value, computed over the entire data set, is also similar for the three methods, with a slight advantage for sensaas with a value of 0.97 Å against 1.06 Å for shaep and 1.09 Å for shafts (Table 2). When considering a RMSD value≤2.0 Å, the number of ligands that are reproduced is still comparable: 1304 with sensaas (89 %), 1305 with shaep (89 %) and 1278 with shafts (87 %). This indicates that it exists at least one ligand structure that allows the correct alignment for nearly all of the ligands. However, when considering only the first, most accurate, RMSD interval [0–0.5], sensaas outperforms the other methods with 941 molecules against 831 and 837 with shaep and shafts, respectively. Table 2 details the number of reproduced ligands and the mean RMSD value for each category. As expected, the number of reproduced ligands decreases according to the difficulty regardless of the method. More than 93 % of ligands are reproduced in the easy and moderate categories, between 75 and 79 % in the hard category and between 31 % and 53 % in the unfeasible category. Consistently, the mean RMSD value for each category increases according to the difficulty. Of note, sensaas generates more accurate results than shaep and shafts in the easy, moderate and hard categories and intermediate results in the unfeasible category. Figure S3 in the Supporting Information details results for the 121 overlays. The average computation time of sensaas is in the order of a few seconds (Table 2). It is longer than that of shaep and shafts (by a factor of 10). sensaas is not fully competitive in its current implementation with established Gaussian‐shape methods in speed. Regarding the surface‐based methods, the computation time of sensaas is comparable to those published by Baum et al.,^20^ which were faster than other surface‐based algorithms.

**Table 2:** Number of reproduced ligands and mean RMSD values for each category for sensaas, shaep and shafts. Average computation time per pairwise alignment is indicated. It takes into account the entire procedure including the calculation of partial charges by cxcalc calculator^49^ for programs shaep and shafts.

		Reproduced ligands (RMSD≤2.0 Å )			Mean RMSD		
Category	Number of ligands	SENSAAS	ShaEP	SHAFTS	SENSAAS	ShaEP	SHAFTS
easy	185	**183 (98.9 %)**	178 (96.2 %)	181 (97.8 %)	**0.37**	0.5	0.46
moderate	991	**927 (93.5 %)**	923 (93.1 %)	922 (93 %)	**0.8**	0.88	0.86
hard	190	148 (77.9 %)	**151 (79.4 %)**	144 (75.8 %)	**1.65**	1.8	1.71
unfeasible	99	46 (46.4 %)	**53 (53.5 %)**	31 (31.3 %)	2.69	**2.58**	3.47
**Total=**	**1465 (100 %)**	1304 (89 %)	**1305 (89 %)**	1278 (87 %)	**0.97**	1.06	1.09
average run time/pairwise alignment		5.7 s	0.51 s	**0.46 s**

### 3.2 Fragment Alignments

**Figure 6:**
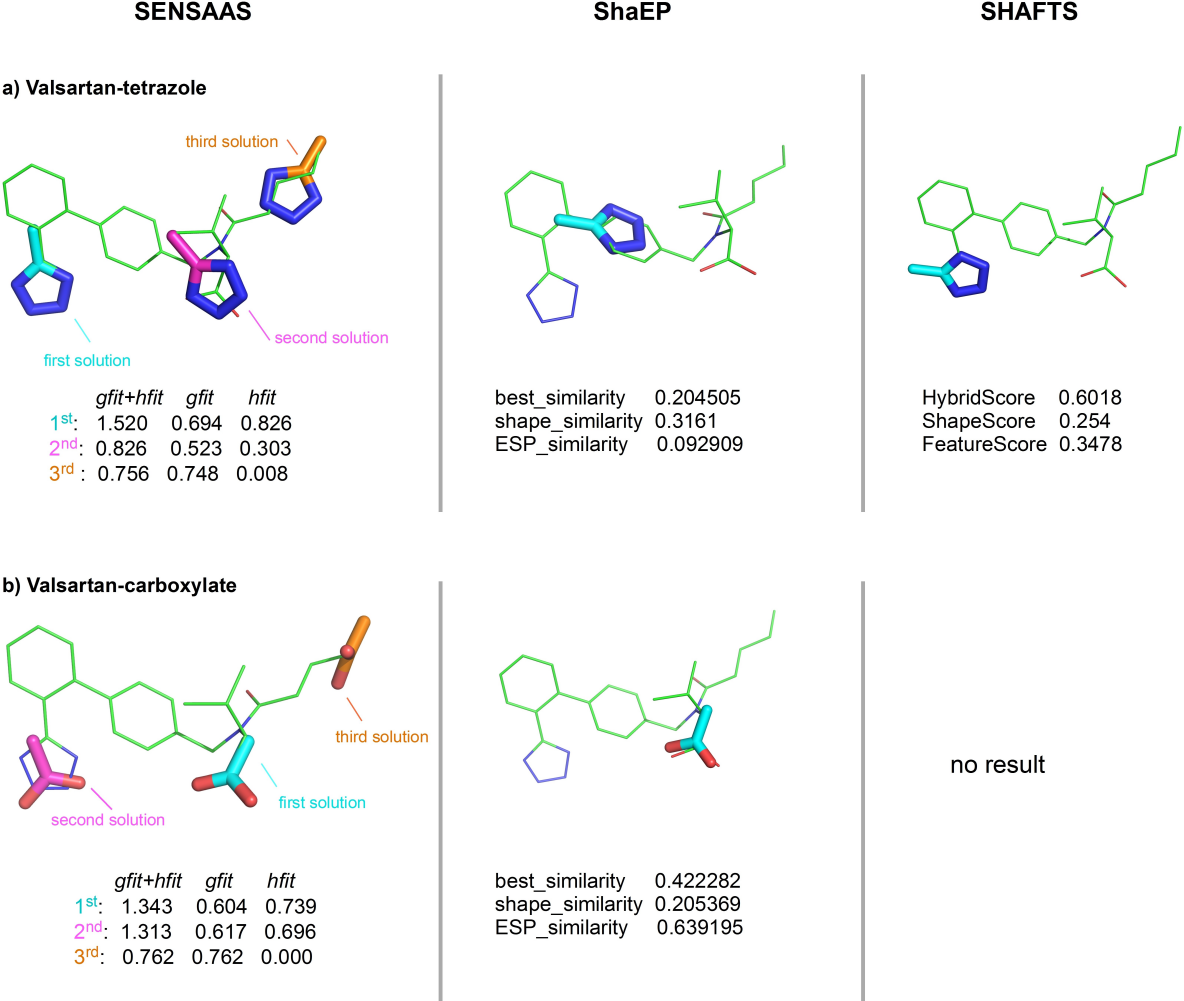
Results of fragment alignments. a) 3D graph superimpositions of Valsartan and tetrazole. b) 3D graph superimpositions of Valsartan and carboxylate. Fitness scores *gfit*, *cfit* and *hfit* are indicated for sensaas. Fitness scores *Best_similarity*, *shape_similarity* and *ESP_similarity* are indicated for shaep. Fitness scores *HybridScore*, *ShapeScore* and *FeatureScore* are indicated for shafts. *Target* 3D structures are colored in green and *Source* 3D structures are colored in cyan (best ranked alignment), in magenta (second ranked alignment) or in orange (third ranked alignment).

In this work, we were particularly interested in the bioisosterism between the tetrazole and the carboxylate function (Figure S1d and e in the Supporting Information). Indeed, although these two functional groups are structurally different, several studies have demonstrated that they may be interchangeable in a bioactive molecule.^46^, ^47^ Figure 6 shows pairwise alignments between a tetrazole or a carboxylate fragment and the drug Valsartan (Figure S1f). As Valsartan contains both tetrazole and carboxylate functions in its structure, alignments with the two fragments are particularly interesting to evaluate possible alternate solutions. Our results show that the expected alignment between the tetrazole fragment and the Valsartan's tetrazole group is ranked first (*gfit+hfit*=1.520) and that two other superimpositions with lower scores are generated: the alignment of the tetrazole fragment with the carboxylate function (*gfit+hfit*=0.826) and with the alkane chain of Valsartan (*gfit+hfit*=0.756) (fragment in cyan, magenta and orange in Figure 6a, respectively). Therefore, three solutions occur: the self‐matching (ranked first), the biosisoteric matching (ranked second), and a geometric matching only with a *hfit* score close to 0 (ranked third). Consistent results are obtained when the carboxylate fragment is aligned on Valsartan. Indeed, the self‐matching is ranked first, the biosisoteric matching is ranked second, and the geometric matching is ranked third with a *hfit* score of 0 (fragment in cyan, magenta and orange in Figure 6b, respectively).

The stochastic nature of sensaas makes it possible to suggest some alternate superimpositions, contrary to deterministic methods that provide only one alignment. As a proof, Figure 6 also displays the alignments generated with shaep and shafts. shaep correctly self‐aligns the carboxylate fragment on Valsartan but not the tetrazole fragment whereas shafts correctly aligns the ring of the tetrazole fragment (apart from the methyl carbon) but not the carboxylate fragment. Actually, these methods do not generate bioisosteric matchings. Similar results were obtained by sensaas when fragments were aligned on the drug Adapalene (Figure S4 in the Supporting Information). In this example, neither shaep nor shafts perfectly aligns fragments on their counterparts. shafts displays a correct alignment for the two oxygens of the carboxylate fragment on Adapalene but not for the methyl carbon perpendicularly oriented.

In summary, these experiments show that sensaas is able to find the correct alignments but also to provide realistic superimpositions by identifying local similarities. Results also show that the algorithm appropriately ranks alternate solutions. This submatching property allows us to envisage more complete studies in the search for bioisosteric replacements.

### 3.3 Discussion

In our approach, a point cloud contains hundreds to thousands colored points, depending on the down‐sampling stage. For a reduced point cloud, between 5 and 95 % of the points are kept when VS ranges from 0.2 to 1.2. This ensures to retain a detailed description of the shape and of the distribution of colors during the alignment. Up to now, the number of input points was a limiting factor in point‐based surface alignment methods. Strategies were used to reduce the number of points by dividing the surfaces into patches, and/or by excluding hydrogen atoms, or by using a united atom model to decrease the number of atoms contributing to the surface. While some of these methods use shape descriptors only,^15^, ^16^ others associate shape and physico‐chemical properties to describe their patches,^17^, ^18^ or use distinct point clouds for molecular shape and for each physico‐chemical property.^19^ Our algorithm, in contrast, uses all atoms and dense point‐based surfaces calculated by the program NSC (600 dots per atomic sphere).^33^ The advantages of using dense point clouds are that a property is not associated with a single point in the space (the center of a patch for example), and that finer variations of physico‐chemical properties can be mapped onto the surface. In the current setting, points are colored according to the pharmacophore feature of their closest atoms: polar, apolar, aromatic and other, but this could easily evolve towards more complexity: additional classes and colors, computed molecular properties such as the partial charge, potential energy… Evaluating the impact of such properties on alignments need to be investigated and assessed.

The present results show that sensaas is able to identify the correct alignment of similar structures (self‐ and substructure matchings) and is able to rank them first. In our approach, the final superimposition strongly relies on a reasonable initial, geometric, alignment obtained with global. This property is consistent with the importance of the shape in molecular interactions and is supported by the performance of methods that only use shape similarities.^1^, ^4^, ^15^, ^16^ However, intermolecular interactions such as electrostatic, hydrophobic or hydrogen bondings must be also taken into account for stabilizing the recognition. Thus, aligning shapes in concert with physico‐chemical features is expected to provide more accurate comparisons between molecules. A strength of our approach is that the initial alignment can be improved by matching both geometric and color information, to get alignments that global could not provide on its own. We also showed that the combination of the scores *gfit* and *hfit* as final selection criterion allows us to discriminate alternate alignments and, specifically, to favor alignments with matching colored features. The general applicability of our alignment method was assessed by using the benchmarking AZ data set. Results showed that sensaas provides accuracy performance equivalent to that of the reference methods shaep and shafts, but also that it generates more accurate alignments in the first precision interval (RMSD≤0.5 Å).

The stochastic nature of point set registration is known. In some usage cases, we would prefer a deterministic approach to always produce the same result, but, we can also take advantage of this stochastic property to suggest several realistic solutions when searching for bioisosteres or scaffold‐hopping, for instance. The example of the alignment of the tetrazole fragment on the drug Valsartan shows that the fragment can match itself but also the carboxylate function as a bioisostere, which was intended.

As a result of this study, the applicability of sensaas is in the field of lead optimization where scaffold hopping and bioisosteric replacement properties of a method are out of importance to identify promising compounds. Indeed, the execution speed of sensaas in its current implementation is prohibitive for virtual screening of large chemical databases. However, even if sensaas is not yet completely optimized and is not fully competitive with established Gaussian‐shape methods in speed, we think that this approach provides a relevant contribution to shape‐based alignment methods.

## 4 Conclusions

Molecular similarity is a central concept in drug discovery. A wide range of methods have been developed to describe molecules and to assess similarity by using representations such as the chemical graph in 2D or 3D, or the molecular shape. In this study, we investigated the use of 3D point set registration methods to identify similarities between colored point‐based molecular surfaces. The resulting workflow, sensaas, was evaluated and validated against several examples, including the benchmarking AZ data set. It shows potential for molecular similarity evaluation, scaffold hopping and bioisosteric replacement. In particular, as it uses open‐source libraries and programs, it can be easily deployed for other pairwise comparison of shapes such as peptides, proteins, cavities (the negative image of the protein surface), or to search for shape complementarity.

## Supplementary Materials

Structure of tested molecules, alignment results of the drug Imatinib with conformers of the substructure Imatinib‐part3, results of the benchmarking AZ data set and results of fragment matching tests.

## Abbreviations

sensaas: sensitive Surface As A Shape
global: Global registration
color: Colored point cloud registration
VS: voxel size
*gfit*: geometric fitness

## Conflict of Interest

None declared.

## Supporting information

As a service to our authors and readers, this journal provides supporting information supplied by the authors. Such materials are peer reviewed and may be re‐organized for online delivery, but are not copy‐edited or typeset. Technical support issues arising from supporting information (other than missing files) should be addressed to the authors.

Supplementary
