# Supplementary material for: sensaas: Shape‐based Alignment by Registration of Colored Point‐based Surfaces
Source: Mol Inform. 2020 Jun 23;39(8):2000081. doi: 10.1002/minf.202000081 (PMC7507133; doi:10.1002/minf.202000081)
Supplement: Supplementary file 1 — Supplementary [file MINF-39-2000081-s001.pdf]

molecular informatics

Supporting Information

SENSAAS: Shape-based Alignment by Registration of Colored Point-based Surfaces

Dominique Douguet* and Frédéric Payan*© 2020 The Authors. Published by Wiley-VCH Verlag GmbH & Co. KGaA. This is an open access article under the terms of the Creative Commons Attribution License, which permits use, distribution and reproduction in any medium, provided the original work is properly cited.

Supporting Information
for

SENSAAS: shape-based alignment by registration of colored point-based surfaces

Dominique Douguet*[a] and Frédéric Payan*[b]

[a] Université Côte d'Azur, Inserm, CNRS, IPMC, 660 route des lucioles 06560 Valbonne, France

[b] Université Côte d'Azur, CNRS, I3S, Les Algorithmes - Euclide B, 2000 route des lucioles 06900 Sophia Antipolis, France

* To whom correspondence should be addressed (douguet@ipmc.cnrs.fr or fpayan@i3s.unice.fr)

Figure S1

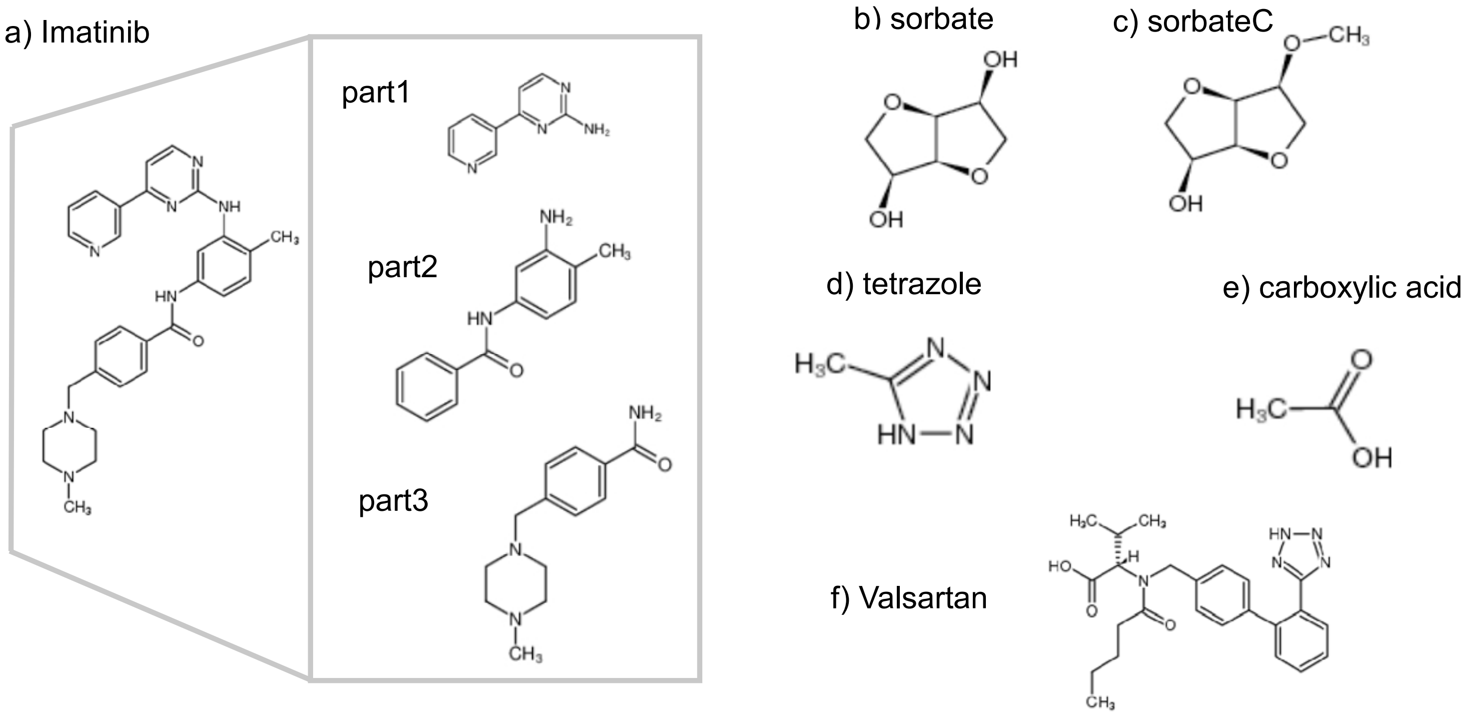

Figure S1 Structure of test molecules. (a) Imatinib drug and three substructures called Imatinib-part1, Imatinib-part2 and Imatinib-part3. (b) small polar molecule sorbate. (c) small polar molecule sorbateC. (d) tetrazole fragment. (e) carboxylic acid fragment. (f) Valsartan drug.

Figure S2

Results of substructure-matching between Imatinib and Imatinib-part3 conformers:

a) Alignment of a conformer of Imatinib-part3 having a RMSD of 1.99 A with the original substructure
Target and *Source* 3D structures are colored in green and cyan, respectively. Hydrogen atoms are hidden.

Imatinib-part3 scores:
gfit+hfit = 1.028
gfit = 0.608
hfit = 0.420

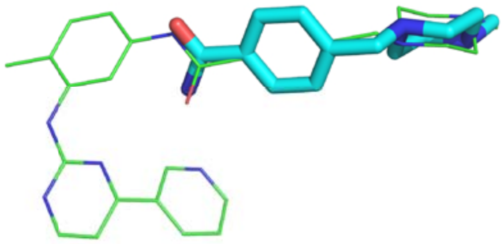

b) *gfit* and *gfit+hfit* values of the alignment of a library of 74 conformers

Conformers were generated using the web server https://chemoinfo.ipmc.cnrs.fr/LEA3D/drawonline.html
It uses the conformer generator RDKit Open-Source Cheminformatics Software with the following options: input structures are read as they are (removeHs=False), the ETKDG method is used to generate conformers that are filtered with a pruneRmsThresh=1, and then, optimized by the MMFF94 force field.

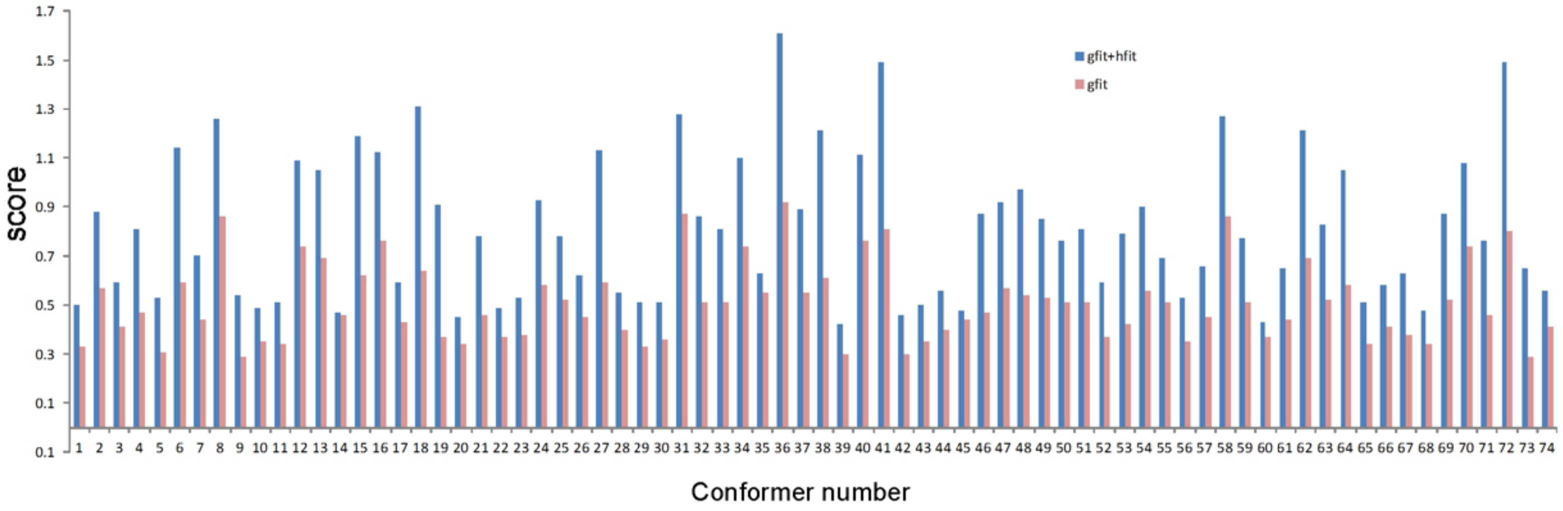

c) Superimposition of the best ranked solution (conformer number 36)
Target and *Source* 3D structures are colored in green and cyan, respectively. Hydrogen atoms are hidden.

Imatinib-part3 scores:
gfit+hfit = 1.610
gfit = 0.920
hfit = 0.690

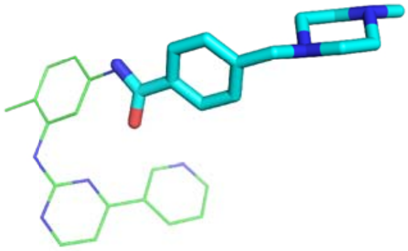

Figure S3

Preparation of ligands of the benchmarking AstraZeneca Data Set

We experimented problems with 6 ligands : P00929-3.sdf, P09955-4.sdf, P43235-1.sdf, Q00511-3.sdf, Q16539-8.sdf and P27487-12.sdf.
Ligand sdf files of P00929-3.sdf, P09955-4.sdf, P43235-1.sdf and Q00511-3.sdf were modified by removing the ‘ M CHG’ line and by setting the atom block related to the charge of the oxygen of the carboxylate function to 0 because a charge of -1 was assigned to the double bonded oxygen in the original sdf file.
Ligand sdf files of Q16539-8.sdf was modified by removing the ‘ M CHG’ line and by setting the atom block related to the charge of the oxygen of the pyridinyloxy to 0 instead of 5.
Ligand P27487-12.sdf was modified by removing an extra bond between atom 48 and 22.

Finally, the first molecule in the set A9JQL9 was separated into two sdf files (A9JQL9-1-1.sdf and A9JQL9-1-2.sdf) because the two ligands are not covalently attached.

cxcalc calculator from JChem Base version 19.19.0 was used for the calculation of partial charges, ChemAxon (http://www.chemaxon.com)

no	Uniprot ID	Name	Family	Organism	Category	Nb ligands	Nb of pairwise calculation except itself	Lowest rmsd values for each ligand SENSAAS	ShaEP	SHAFTS
1	P00374	dihydrofolate reductase	oxidoreductase	Homo Sapiens	easy	15	210	0.281	0.289	0.317
2	P00489	protein (glycogen phosphorylase)	transferase	Oryctolagus Cuniculus	easy	20	380	0.135	0.136	0.122
3	P00509	aspartate aminotransferase	transferase	Escherichia Coli	easy	6	30	0.297	0.898	0.433
4	P00797	renin	hydrolase	Homo Sapiens	easy	5	20	0.314	0.264	0.366
5	P05326	Isopenicillin n synthase	oxidoreductase	Emericella Nidulans	easy	9	72	0.241	0.266	0.249
6	P07688	cathepsin B	hydrolase	Bos Taurus	easy	7	42	0.184	1.059	0.231
7	P08235	mineralocorticoid receptor	none	Homo Sapiens	easy	7	42	0.703	0.771	0.347
8	P0A017	dihydrofolate reductase	oxidoreductase	Staphylococcus Aureus	easy	11	110	0.262	0.700	0.320
9	P0ABP9	purine nucleoside phosphorylase	transferase	Escherichia Coli	easy	8	56	0.391	0.345	0.415
10	P12758	uridine phosphorylase	transferase	Escherichia Coli	easy	5	20	0.298	0.354	1.502
11	P16184	dihydrofolate reductase	oxidoreductase	Pneumocystis Carinii	easy	7	42	0.443	0.504	0.501
12	P22906	dihydrofolate reductase	oxidoreductase	Candida Albicans	easy	8	56	0.200	0.261	0.215
13	P23470	receptor-type tyrosine-protein phosphatase gamma	hydrolase	Homo Sapiens	easy	5	20	0.218	0.308	0.254
14	P30291	wee1-like protein kinase	transferase	Homo Sapiens	easy	7	42	0.217	0.267	0.220
15	P36897	TGF-beta receptor type I	transferase	Homo Sapiens	easy	5	20	0.552	0.524	0.536
16	P51955	serine/threonine-protein kinase NEK2	transferase	Homo Sapiens	easy	10	90	0.267	0.609	0.290
17	P54760	ephrin type-B receptor 4	transferase	Homo Sapiens	easy	9	72	0.188	0.181	0.244
18	P56658	adenosine deaminase	hydrolase	Bos Taurus	easy	9	72	0.444	0.851	1.004
19	P61823	pancreatic ribonuclease A	hydrolase	Bos Taurus	easy	7	42	1.324	1.413	1.251
20	Q10714	angiotensin converting enzyme	hydrolase	Drosophila Melanogaster	easy	6	30	0.362	0.370	0.347
21	Q57834	tyrosyl-tRNA synthetase	ligase	Methanocaldococcus Jannaschii	easy	7	42	0.454	0.451	0.491
22	Q9BJF5	calmodulin-domain protein kinase 1	transferase	Toxoplasma Gondii	easy	12	132	0.283	0.344	0.508
23	O14757	serine/threonine-protein kinase Chk1	transferase	Homo Sapiens	moderate	35	1190	0.397	0.532	0.354
24	O14965	serine/threonine-protein kinase 6	transferase	Homo Sapiens	moderate	11	110	1.389	1.690	0.788
25	O15530	3-phosphoinositide dependent protein kinase-1	transferase	Homo Sapiens	moderate	14	182	1.214	0.637	0.392
26	O60674	tyrosine-protein kinase JAK2	transferase	Homo Sapiens	moderate	13	156	0.494	0.755	0.618
27	O60885	human BRD4	none	Homo Sapiens	moderate	10	90	0.368	0.430	0.563
28	O76074	cGMP-specific 3',5'-cyclic phosphodiesterase	hydrolase	Homo Sapiens	moderate	9	72	0.652	0.678	0.783
29	O76290	pteridine reductase	oxidoreductase	Trypanosoma Brucei Brucei	moderate	8	56	1.360	1.203	1.100
30	P00469	thymidylate synthase	transferase	Lactobacillus Casei	moderate	5	20	2.548	3.360	2.552
31	P00517	cAMP-dependent protein kinase, alpha-catalytic subunit	transferase	Bos Taurus	moderate	21	420	0.528	0.647	0.814
32	P00520	proto-oncogene tyrosine-protein kinase ABL	transferase	Mus Musculus	moderate	5	20	1.430	1.884	3.296
33	P00523	proto-oncogene tyrosine-protein kinase Src	transferase	Gallus Gallus	moderate	12	132	1.845	2.432	1.069
34	P00730	carboxypeptidase A	hydrolase	Bos Taurus	moderate	8	56	0.771	0.693	0.743
35	P00734	alpha thrombin	hydrolase	Homo Sapiens	moderate	28	756	0.249	0.306	0.333
36	P00742	coagulation factor XA	hydrolase	Homo Sapiens	moderate	37	1332	0.239	0.300	0.332
37	P00749	protein (urokinase-type plasminogen activator)	hydrolase	Homo Sapiens	moderate	27	702	0.403	0.453	0.398
38	P00760	trypsin	hydrolase	Bos Taurus	moderate	22	462	0.243	0.313	0.263
39	P00918	carbonic anhydrase II	lyase	Homo Sapiens	moderate	23	506	0.287	0.401	0.299
40	P00929	tryptophan synthase	lyase	Salmonella Typhimurium	moderate	10	90	0.673	0.537	0.477
41	P02829	HSP82	none	Saccharomyces Cerevisiae	moderate	11	110	0.485	1.113	0.463
42	P03372	oestrogen receptor	none	Homo Sapiens	moderate	27	702	0.387	0.310	0.395
43	P04035	protein (HMG-COA reductase)	oxidoreductase	Homo Sapiens	moderate	7	42	0.241	0.849	0.223
44	P04642	L-lactate dehydrogenase A chain	oxidoreductase	Rattus Norvegicus	moderate	8	56	0.990	1.059	1.631
45	P06401	progesterone receptor	none	Homo Sapiens	moderate	9	72	0.861	0.414	1.160
46	P07900	HSP 90-alpha	hydrolase	Homo Sapiens	moderate	23	506	0.263	0.383	0.296
47	P08069	insulin-like growth factor 1 receptor precursor	transferase	Homo Sapiens	moderate	8	56	1.551	2.213	2.378
48	P08254	stromelysin-1	hydrolase	Homo Sapiens	moderate	9	72	1.188	0.661	1.292
49	P08581	hepatocyte growth factor receptor	transferase	Homo Sapiens	moderate	13	156	1.012	1.173	0.875
50	P08709	coagulation factor VII	hydrolase	Homo Sapiens	moderate	5	20	0.686	3.224	0.628
51	P09467	fructose-1,6-bisphosphatase 1	hydrolase	Homo Sapiens	moderate	6	30	0.382	0.365	0.387
52	P09955	procarboxypeptidase B	hydrolase	Sus Scrofa	moderate	9	72	0.316	0.249	0.320
53	P09960	leukotriene A-4 hydrolase	hydrolase	Homo Sapiens	moderate	19	342	0.587	0.287	0.497
54	P0A5J2	methionine aminopeptidase	hydrolase	Mycobacterium Tuberculosis	moderate	6	30	0.505	1.845	1.292
55	P0AD64	beta-lactamase SHV-1	hydrolase	Klebsiella Pneumoniae	moderate	6	30	1.922	2.767	2.263
56	P0AE18	Methionine aminopeptidase	hydrolase	Escherichia Coli	moderate	21	420	0.479	0.576	0.557
57	P0C5C1	beta-lactamase	hydrolase	Mycobacterium Tuberculosis	moderate	8	56	0.459	1.268	2.136
58	P10275	androgen receptor	none	Homo Sapiens	moderate	11	110	0.344	0.362	0.713
59	P11509	cytochrome P450, family 2, subfamily A, polypeptide 6	oxidoreductase	Homo Sapiens	moderate	6	30	2.103	1.260	1.564
60	P14324	farnesyl pyrophosphate synthetase	transferase	Homo Sapiens	moderate	8	56	0.310	0.405	0.460
61	P15090	fatty acid-binding protein, adipocyte	none	Homo Sapiens	moderate	8	56	0.651	0.734	1.120

62	P15121	aldose reductase	oxidoreductase	Homo Sapiens	moderate	27	702	0.600	0.793	1.074
63	P17612	cAMP-dependent protein kinase	transferase	Homo Sapiens	moderate	13	156	0.702	1.273	0.498
64	P18031	protein (protein-tyrosine phosphatase 1b)	hydrolase	Homo Sapiens	moderate	30	870	0.495	0.500	0.349
65	P24182	biotin carboxylase	ligase	Escherichia Coli	moderate	12	132	1.298	0.648	0.629
66	P24941	cyclin-dependent kinase 2	transferase	Homo Sapiens	moderate	24	552	0.463	0.610	0.480
67	P25440	bromodomain-containing protein 2	none	Homo Sapiens	moderate	11	110	0.646	0.566	1.135
68	P25774	cathepsin S	hydrolase	Homo Sapiens	moderate	14	182	0.326	0.422	0.504
69	P25779	cruzain	hydrolase	Trypanosoma Cruzi	moderate	7	42	1.659	1.477	1.041
70	P27487	dipeptidyl peptidase IV soluble form	hydrolase	Homo Sapiens	moderate	39	1482	0.344	0.460	0.411
71	P28482	mitogen-activated protein kinase 1	transferase	Homo Sapiens	moderate	7	42	0.963	1.024	1.306
72	P28845	corticosteroid 11-beta-dehydrogenase isozyme 1	oxidoreductase	Homo Sapiens	moderate	9	72	1.004	1.103	1.876
73	P30405	peptidyl-prolyl cis-trans isomerase F, mitochondrial	isomerase	Homo Sapiens	moderate	5	20	1.992	2.080	1.823
74	P35557	glucokinase isoform 2	transferase	Homo Sapiens	moderate	7	42	1.591	0.867	0.990
75	P35968	vascular endothelial growth factor receptor 2	transferase	Homo Sapiens	moderate	8	56	0.498	0.504	1.226
76	P39900	macrophage metalloelastase	hydrolase	Homo Sapiens	moderate	17	272	0.514	0.685	0.763
77	P41148	endoplasmin	none	Canis Lupus Familiaris	moderate	8	56	0.668	1.183	0.680
78	P43235	cathepsin K	hydrolase	Homo Sapiens	moderate	13	156	0.985	0.677	0.786
79	P45452	collagenase 3	hydrolase	Homo Sapiens	moderate	12	132	0.434	0.667	0.541
80	P47811	mitogen-activated protein kinase 14	transferase	Mus Musculus	moderate	10	90	0.612	0.497	0.595
81	P51857	3-oxo-5-beta-steroid 4-dehydrogenase	oxidoreductase	Homo Sapiens	moderate	6	30	0.632	0.942	1.107
82	P56817	beta-secretase 1	hydrolase	Homo Sapiens	moderate	18	306	0.626	0.761	0.624
83	P78536	ADAM 17	hydrolase	Homo Sapiens	moderate	15	210	1.157	1.305	0.466
84	Q00511	uricase	oxidoreductase	Aspergillus Flavus	moderate	8	56	0.684	0.534	0.943
85	Q02127	dihydroorotate dehydrogenase, mitochondrial	oxidoreductase	Homo Sapiens	moderate	8	56	0.388	0.403	0.433
86	Q04771	activin receptor type-1	transferase	Homo Sapiens	moderate	5	20	0.374	0.556	0.396
87	Q07343	cAMP-specific 3',5'-cyclic phosphodiesterase 4B	hydrolase	Homo Sapiens	moderate	14	182	0.446	0.489	0.561
88	Q13526	peptidyl-prolyl cis-trans isomerase NIMA- Interacting 1	isomerase	Homo Sapiens	moderate	23	506	0.460	0.579	0.563
89	Q16539	p38 MAP kinase	transferase	Homo Sapiens	moderate	27	702	0.443	0.493	0.430
90	Q581W1	pteridine reductase 1	oxidoreductase	Trypanosoma Brucei Brucei	moderate	9	72	0.746	0.380	0.338
91	Q92731	estrogen receptor beta	none	Homo Sapiens	moderate	18	306	0.488	0.391	0.602
92	Q9L5C8	beta-lactamase CTX-M-9	hydrolase	Escherichia Coli	moderate	14	182	0.961	0.794	0.933
93	Q9QYJ6	phosphodiesterase-10A	hydrolase	Rattus Norvegicus	moderate	10	90	0.894	0.602	0.820
94	Q9T0N8	cytokinin dehydrogenase 1	oxidoreductase	Zea Mays	moderate	8	56	1.473	0.408	0.784
95	Q9Y233	cAMP and cAMP-inhibited cGMP 3', 5'-cyclic phosphodiesterase 10A	hydrolase	Homo Sapiens	moderate	9	72	1.753	1.391	1.800
96	O15530_allosteric		transferase	Homo Sapiens	hard	5	20	1.692	1.174	2.836
97	P00772	elastase	hydrolase	Sus Scrofa	hard	5	20	2.194	3.148	3.148
98	P00808	beta-lactamase	hydrolase	Bacillus Licheniformis	hard	8	56	2.584	2.600	2.166
99	P06239	LCK kinase	transferase	Homo Sapiens	hard	10	90	0.957	2.513	1.354
100	P11309	proto-oncogene serine/threonine-protein kinase Pim-1	transferase	Homo Sapiens	hard	31	930	0.912	0.910	0.899
101	P14324_allosteric		transferase	Homo Sapiens	hard	7	42	1.340	2.119	1.234
102	P28523	casein kinase II	transferase	Zea Mays	hard	19	342	1.054	0.738	0.849
103	P42330	aldo-keto reductase family 1 member C3	oxidoreductase	Homo Sapiens	hard	10	90	1.662	1.403	2.166
104	P48736	phosphatidylinositol-4,5-bisphosphate 3-kinase	transferase	Homo Sapiens	hard	5	20	0.854	3.710	2.412
105	P49841	glycogen synthase kinase-3 beta	transferase	Homo Sapiens	hard	13	156	1.284	1.001	0.928
106	P50579	protein (methionine aminopeptidase)	hydrolase	Homo Sapiens	hard	9	72	1.220	1.862	1.431
107	P52700	metallo-beta-lactamase L1	hydrolase	Stenotrophomonas Maltophilia	hard	6	30	0.948	1.352	1.022
108	P53779	mitogen-activated protein kinase 10	transferase	Homo Sapiens	hard	16	240	0.881	0.726	0.907
109	P68400	casein kinase II	transferase	Homo Sapiens	hard	14	182	1.634	1.503	1.871
110	P80457	xanthine dehydrogenase	oxidoreductase	Bos Taurus	hard	9	72	1.850	2.086	1.580
111	Q08499	cAMP-specific 3',5'-cyclic phosphodiesterase 4D	hydrolase	Homo Sapiens	hard	14	182	0.676	0.558	0.650
112	Q3JRA0	2-C-methyl-D-erythritol 2,4-cyclodiphosphate synthase	lyase	Burkholderia Pseudomallei	hard	4	12	4.705	4.390	4.605
113	Q9BZP6	acidic mammalian chitinase	hydrolase	Homo Sapiens	hard	5	20	3.322	0.780	0.850
114	A9JQL9	dehydrosqualene synthase	transferase	Staphylococcus Aureus	unfeasible	7	42	2.723	2.867	4.833
115	P00811	beta-lactamase	hydrolase	Escherichia Coli	unfeasible	22	462	1.638	1.315	1.733
116	P04058	acetylcholinesterase	hydrolase	Torpedo Californica	unfeasible	8	56	2.219	1.938	3.345
117	P11838	endothiapepsin	hydrolase	Cryphonectria Parasitica	unfeasible	11	110	1.978	2.413	4.134
118	P14174	macrophage migration inhibitory factor	isomerase	Homo Sapiens	unfeasible	16	240	2.154	2.300	2.734
119	P24627	lactotransferrin	hydrolase	Bos Taurus	unfeasible	12	132	3.439	2.857	4.210
120	P42574	caspase-3	hydrolase	Homo Sapiens	unfeasible	7	42	4.036	3.596	3.664
121	P59071	phospholipase A2	hydrolase	Daboia Russellii Pulchella	unfeasible	16	240	3.368	3.403	3.134

Figure S4

Results of fragment alignments

a) Structure of the drug Adapalene

Adapalene

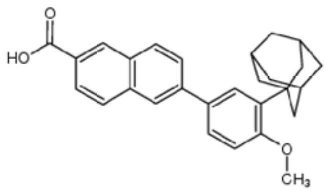

b) 3D graph superimpositions of Adapalene / carboxylate and Adapalene / tetrazole

For SENSAAS, 100 runs were performed. Each run was started with a random orientation for Source. Fitness scores *gfit+hfit*, *gfit* and *hfit* are indicated.
For ShaEP, fitness scores best_similarity, shape_similarity and ESP_similarity are indicated.
For SHAFTS, fitness scores HybridScore, ShapeScore and FeatureScore are indicated.
Target 3D structures are colored in green and Source 3D structures are colored in cyan (best ranked alignment), in magenta (second ranked alignment) or in orange (third ranked alignment).

SENSAAS

Adapalene-carboxylate

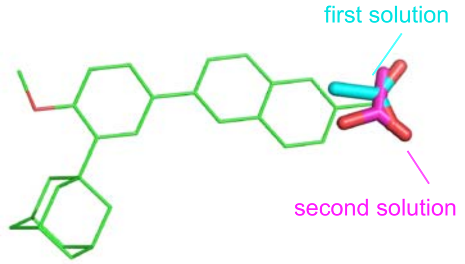

	gfit+hfit	*gfit*	*hfit*
1st :	1.525	0.685	0.840
2nd:	1.132	0.622	0.510

Adapalene-tetrazole

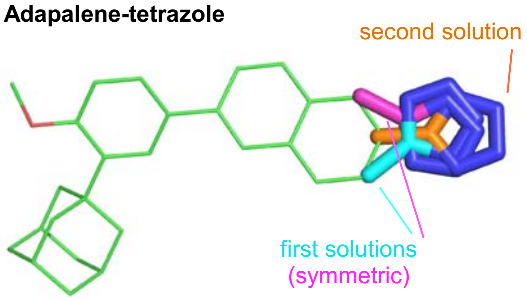

	gfit+hfit	*gfit*	*hfit*
1st and 2nd:	1.310	0.661	0.649
3rd:	1.111	0.551	0.560

ShaEP

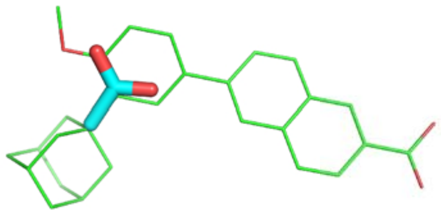

best_similarity	0.172882
shape_similarity	0.239606
ESP_similarity	0.106158

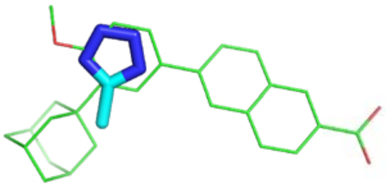

best_similarity	0.450079
shape_similarity	0.312891
ESP_similarity	0.587267

SHAFTS

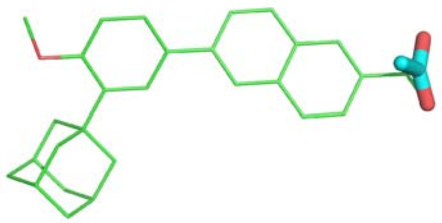

HybridScore	0.4684
ShapeScore	0.131
FeatureScore	0.3374

no result
